# Plant IsomiR Atlas: Large Scale Detection, Profiling, and Target Repertoire of IsomiRs in Plants

**DOI:** 10.3389/fpls.2018.01881

**Published:** 2019-01-22

**Authors:** Kun Yang, Xiaopeng Wen, Suresh B. Mudunuri, Gaurav Sablok

**Affiliations:** ^1^Key Laboratory of Plant Resources Conservation and Germplasm Innovation in Mountainous Region (Guizhou University), Ministry of Education, Institute of Agro-Bioengineering, College of Life Sciences, Guizhou University, Guiyang, China; ^2^Centre for Bioinformatics Research, SRKR Engineering College, Bhimavaram, India; ^3^Finnish Museum of Natural History, University of Helsinki, Helsinki, Finland; ^4^Organismal and Evolutionary Biology (OEB) Research Programme, Department of Biological and Environmental Sciences, University of Helsinki, Helsinki, Finland

**Keywords:** isomiRs, microRNAs, plants, post-transcriptional machinery, functional targets

## Abstract

microRNAs (miRNAs) play an important role as key regulators controlling the post-transcriptional events in plants across development, abiotic and biotic stress, tissue polarity and also in defining the evolutionary basis of the origin of the post-transcriptional machinery. Identifying patterns of regulated and co-regulated small RNAs, in particular miRNAs and their sequence variants with the availability of next generation sequencing approaches has widely demonstrated the role of miRNAs and their temporal regulation in maintaining plant development and their response to stress conditions. Although the role of canonical miRNAs has been widely explored and functional diversity is revealed, those works for isomiRs are still limited and urgent to be carried out across plants. This relative lack of information with respect to isomiRs might be attributed to the non-availability of large-scale detection of isomiRs across wide plant species. In the present research, we addressed this by developing Plant isomiR Atlas, which provides large-scale detection of isomiRs across 23 plant species utilizing 677 smallRNAs datasets and reveals a total of 98,374 templated and non-templated isomiRs from 6,167 precursors. Plant isomiR Atlas provides several visualization features such as species specific isomiRs, isomiRs and canonical miRNAs overlap, terminal modification classifications, target identification using psRNATarget and TargetFinder and also canonical miRNAs:target interactions. Plant isomiR Atlas will play a key role in understanding the regulatory nature of miRNAome and will accelerate to understand the functional role of isomiRs. Plant isomiR Atlas is available at www.mcr.org.in/isomir.

**One Sentence Summary**

Plant isomiR Atlas will play a key role in understanding the regulatory nature of miRNAome and will accelerate the understanding and diversity of functional targets of plants isomiRs.

## Introduction

Mechanistic understanding of plant RNA biology and identifying classes and patterns of smallRNAs, a class of non-coding RNAs has evolved particularly after the advent of the next-generation sequencing. Non-coding RNAs such as microRNAs, long non-coding RNAs (lncRNAs), miRtrons (miRNAs located in introns), artificial miRNAs, siRNAs, phasiRNAs, and trans-acting siRNAs play an important role in regulating the post-transcriptional responses at various levels, such as at different stages of development in response to abiotic and biotic stress (Lin et al., 2018) or by acting as targets for endogenous silencing mechanism (Neilsen et al., 2012; Sablok et al., 2015). Accelerated application of next generation sequencing techniques has revealed not only the concordance and dis-concordant patterns of miRNA regulation but has also enabled the identification and discovery of the miRNA variants, binding site efficiency, phase shifting and phasiRNAs (Yang et al., 2018). Fundamental application of miRNAs either from the sequence conservation or from the binding site conservation viewpoint has unfolded conserved regulatory relation accross plants, e.g. members from MIR156 target SQUAMOSA promoter binding protein-like (SPL) genes in plants which involved in development (Stief et al., 2014). Nonetheless, recently explored miRNA derived siRNAs piggybacking for virus resistance (Incarbone et al., 2018) can be widely exploited for developing miRNA-based approaches for plant domestication.

After the first report of the microRNAs canonical variants (isomiRs) in *Oryza sativa* (Morin et al., 2008a), these canonical variants (isomiRs) have been shown to play an important role in regulating the post-transcriptional responses in response to several abiotic and biotic responses (Gozmanova et al., 2017) and recently much emphasis has been laid on the discovery of these variants to unravel the biogenesis pathway and target potential of these isomiRs. Relative features such as length variations have classified these isomiRs into several classes, which includes 5′- isomiRs, 3′- isomiRs, polymorphic isomiRs, template, and non-templated isomiRs (Neilsen et al., 2012; Rogans and Rey, 2016). Plant isomiR biogenesis pathway has not been widely studied, however, several reports hypothesize the ribonuclease DICER-LIKE 1 (DCL) cleavage variations as the prominent cause of isomiR biogenesis (Ruby et al., 2006; Landgraf et al., 2007; Kuchenbauer et al., 2008; Morin et al., 2008a,b). For non-templated isomiRs, their origin is thought as coming from nucleotidyltransferase like PAPD4 (Burroughs et al., 2010) describing the addition of templated or non-templated nucleotides to isomiRs terminus. It has been previously shown that some of the observed substitutions, with few relative occurrences within the internal sites may be an effect of post-transcriptional RNA editing (Neilsen et al., 2012; Rogans and Rey, 2016). Although uridylation events have been primarily linked to the terminal modification or additions leading to the biogenesis of isomiRs, concluding evidences reveal that the complete biogenesis pathway remains a challenging question.

Irrespective of its biogenesis pathway, target site accessibility and whether or not these isomiRs play a role in expanding the target predictions has been recently addressed on a large scale in model plant species (Ahmed et al., 2014). Ahmed et al. (2014) performed comprehensive analysis of isomiR terminal heterogeneity in demonstrating the effect on the accessibility of target binding demonstrating that combination of miRNAs and isomiRs increased the accuracy of target prediction in *A. thaliana* (Ahmed et al., 2014). Additionally, several targets were shared by members of isomiRs and they were enriched for certain GO terms (Ahmed et al., 2014). This is further evident from the recent reports in *Phaseoleae*, where target binding conversation and enhancement was seen in canonical miRNA and terminal isoforms of the miR1510 family (Fei et al., 2018). Furthermore, many targets of isomiRs were also targets of canonical miRNAs indicating that miRNA-mediated gene regulation were also assisted by isomiRs (Ahmed et al., 2014).

Terminal modifications, which can be addition or substitution events have been primarily defined as an essential criteria for the classification of isomiRs and have been widely used as a. golden rule to identify and classify isomiRs by several tools such as isomiRID (de Oliveira et al., 2013), isomiRage (Muller et al., 2014), DeAnnoIso (Zhang et al., 2016), miR-isomiRExp (Guo et al., 2016), and isomiR2Function (Yang et al., 2017). Although the above-mentioned tools present a classification approach for the identification of isomiRs, large-scale identification and functional repertoire of these isomiRs across plant species and their cross-conservation of isomiRs and their corresponding targets present a knowledge gap that is yet to be addressed. In plants, recently developed isomiRBank (Zhang et al., 2016) represents one such repository, which displays isomiRs detection across 4 plant species and lacks the large-scale detection of isomiRs and their corresponding classification and target diversity taking into account the terminal modifications as described previously (Neilsen et al., 2012; Rogans and Rey, 2016). In plants, preferential association of the miRNAs with the AGO has been widely demonstrated and well described (Fátyol et al., 2015; Incarbone et al., 2018). miR390 presents an classical example of AGO divergence, which has preference for loading on AGO7 for the production of the TAS3 ta-siRNAs (Axtell et al., 2006; Incarbone et al., 2018) but has also shown to be loading on AGO2 due to its 5′A, which assists in the AGO2 loading (Mi et al., 2008) thus demonstrating that the terminal nucleotides at the 5′ or 3′ can influence the preferential AGO selection. With the limited knowledge about the diversity and distribution of the terminal nucleotides in isomiRs with respect to the canonical miRNA, it is still a challenging task to describe the preferential association of the isomiRs to AGO and the preferential terminal nucleotides which affect the miRNA+isomiR target abundance.

Additionally, the role of the isomiRs generated from the canonical miRNAs and the target mimics has not been defined. Moreover, it is worth highlighting that previously developed visualization approaches such as isomiRBank have largely focused on the detection of templated isomiRs, which presents a gap in understanding of non-templated isomiRs in plants. To address this knowledge gap, such as (1) low number of species present in isomiRBank (Zhang et al., 2016), (2) absence of the non-templated isomiR repository, (3) targets of the detected isomiRs, (4) consensus target calling through the implementation of several target prediction approaches and (5) isomiRs and target interactions including target mimics, we present Plant isomiR Atlas (www.mcr.org.in/isomir), which is a large scale repository of plant specific isomiRs across 23 pecies representing a total of 677 datasets and presents both templated and non-templated isomiRs profiling with support for two target prediction algorithms and target mimics. We believe that the profiled isomiRs and the ease of visualization of the isomiRs along with the target classification and miRNA mimic under one common framework will accelerate the functional discovery of isomiRs and will increase the targeted miRNA'ome in plants.

## Materials and Methods

### Data Resources

Genome datasets with corresponding features in GFF3 format, annotations, and transcripts were downloaded for 23 plant species: *Amborella trichopoda, Arabidopsis lyrata, Arabidopsis thaliana, Brachypodium distachyon, Brassica rapa, Carica papaya, Citrus clementina, Citrus sinensis, Glycine max, Gossypium raimondii, Malus domestica, Manihot esculenta, Medicago truncatula, Nelumbo nucifera, Oryza sativa, Populus trichocarpa, Setaria italic, Solanum lycopersicum, Solanum tuberosum, Sorghum bicolor, Triticum aestivum, Vitis vinifera*, and *Zea mays* from Phytozome version 11 accessible through www.phytozome.net/ (Goodstein et al., 2011). smallRNA datasets relevant to these species were identified through curated literature searches and their information can be found in Table S1. For the aforementioned species, pre-miRNAs and mature miRNAs corresponding to these species were downloaded from miRBase release 21 accessible through http://www.mirbase.org (Kozomara and Griffiths-Jones, 2013) and plant microRNA database (PMRD) accessible through: bioinformatics.cau.edu.cn/PMRD (Zhang et al., 2009). tRNAs and rRNAs were downloaded from RFAM (http://rfam.xfam.org/), plant snoRNAs are available from (http://bioinf.scri.sari.ac.uk/cgi-bin/plant_snorna/home).

### Large Scale Detection of Plant IsomiRs, Targets, and Endogenous miRNAs

**Figure 1:**
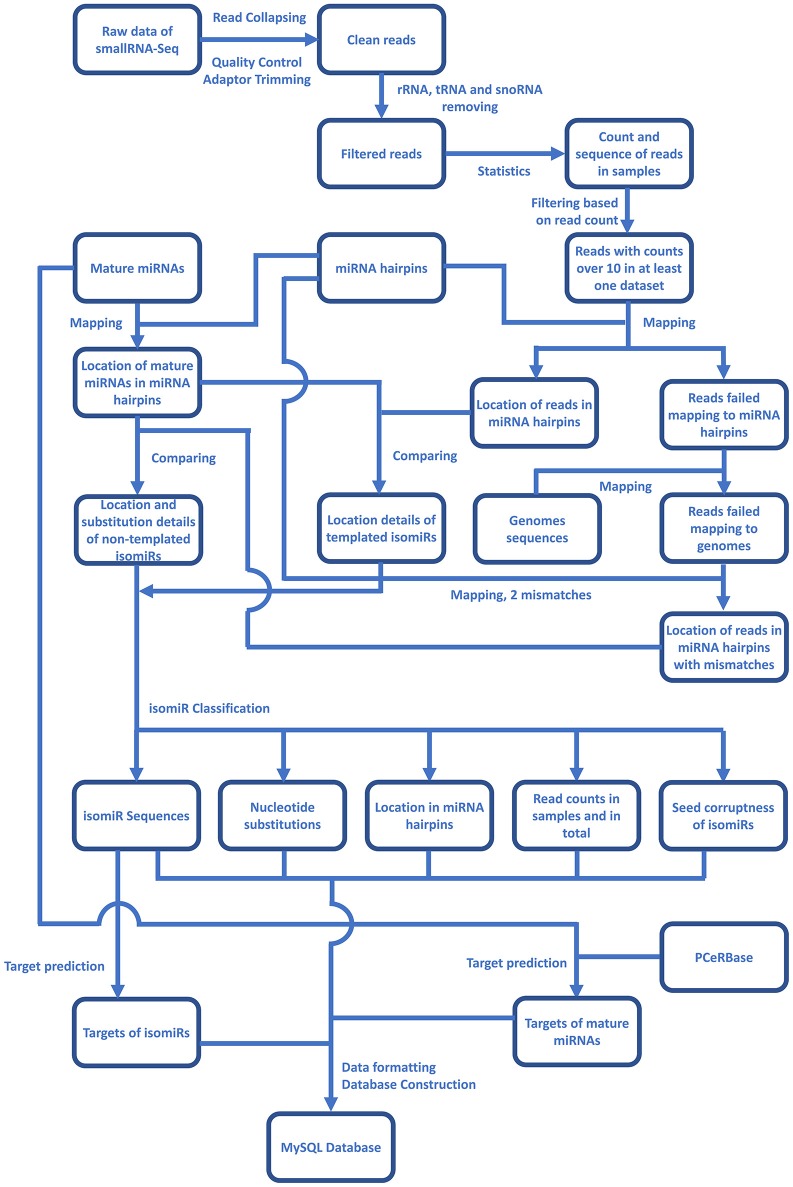
An overlay of the bioinformatics workflow in Plant isomiR Atlas. Mapping reads on genome and miRNA precursors were carried out by Bowtie1. isomiRs of both types were identified and classified in details by algorithm of isomiR2Function (Yang et al., 2017).

For the identification of plant isomiRs, quality control, and adaptor trimming were done by a perl script which calls cutadapt (Martin, 2011) and indicates counts of high-quality reads. All the clean reads were further mapped to RFAM (http://rfam.xfam.org/), tRNAs (http://rfam.xfam.org/), and plant snoRNAs database (http://bioinf.scri.sari.ac.uk/cgi-bin/plant_snorna/home) to remove the background noise coming from other RNA classes. Reads with read count lower than 10 were also removed from further analysis to avoid reads generated by any sequencing error or representing the low coverage reads. The entire bioinformatic workflow for Plant isomiR Atlas is described in Figure 1, which outlines the detection of templated and non-templated isomiRs. Briefly, to identify templated isomiRs, remain reads were mapped on miRNA hairpins of corresponding species allowing no mismatch. Reads successfully mapped to hairpins were defined as templated isomiRs. For reads failed mapping to any miRNA hairpin, they were mapped to genome allowing no mismatch to make sure they were not generated from other place in genome. Among them, reads failed mapping to the genome were then mapped to miRNA hairpins again allowing two mismatches. Reads successfully mapped to hairpins were defined as non-templated isomiRs. After templated and non-templated isomiRs had been identified, they were classified in three aspects: location, substitution and seed corruptness. More details on isomiR identification can be found at the Method page of the database (http://www.mcr.org.in/isomir/method.php?pg=2). We classified isomiRs in three aspects: location, substitution and seed corruptness. Substitution patterns have been defined as 5 V for substitutions happened at 5′ terminus, 3 V for substitution happened at 3′ terminus, TS for substitutions happened internally and tandemly, MS for substitutions happened internally and randomly, SS for single substitutions happened internally, CV for substitutions happened terminally and internally. Location patterns have been defined as non-drift for isomiRs generated from the same loci that generates canonical miRNAs. Additionally, [5′/3′][+/–] have been defined for those isomiRs whose 5′ or 3′ terminal position is upstream or downstream of the corresponding canonical miRNAs, e.g., 5′+3 indicates that isomiR has three additional bases at its 5′ terminus compared to canonical miRNAs. Seed corruptness patterns have been defined as [F/f] for whether the similar seed sequence was between 2 and 8 bases and [C/c] for whether the seed sequence was the same with overlapped canonical miRNA's. Since isomiRs shows sequence difference to the canonical miRNA, all the identified non-drift isomiRs are non-templated, which are defined on the basis of the internal SNPs counted as mismatches against miRNA hairpin.

Taking into account the differences in the target prediction algorithms, we implemented two target prediction tools, psRNATarget (Dai and Zhao, 2011) and TargetFinder (Fahlgren et al., 2007) with default parameters. For the identification of the miRNA-target interactions, all the miRNAs-target interactions were downloaded from PCeRBase (Yuan et al., 2017) including the target mimics interactions. Since we aimed toward the identification of the isomiRs spanning across the datasets, the read normalization was therefore not done as the proposed goal of the Plant isomiR Atlas is large scale detection of isomiRs and read normalization can bias the detection of the isomiRs, where each of the datasets embedded may have different read counts in the respective experiments. Plant isomiR Atlas has been developed using the MySQL as a backend database and uses PHP as a front end. Plant isomiR Atlas is hosted on a 16 core Intel Xeon machine with Ubuntu as an operating system and allows for the rapid searches and easy to browse interface is equipped with several search options.

## Results and Discussion

### High Throughput IsomiR Profiling Across Plant Clade

Identification and profiling of isomiRs has recently gained importance taking into account the increasing reports of isomiRs present in plants genomes and their consecutive involvement in response to abiotic stresses (Ahmed et al., 2014; Sablok et al., 2015). Several genomics and bioinformatics approaches have been developed, however large-scale mining of isomiRs and their relationship with canonical miRNAs and corresponding target divergence and conversation are some of the questions which demand attention to understand the origin and diversity of isomiRs. Plant isomiR Atlas fills this knowledge gap by providing large-scale isomiRs across 23 species. High throughput profiling of isomiRs across plant genomes using the criteria (see Material and Methods) allowed the detection of unique 98,374 templated and non-templated isomiRs from 6,167 precursors, with species *G. max* (13,478), *O. sativa* (24,503), *A. thaliana* (17,516), and *M. truncatula* (7,584) showing the maximum number of unique isomiRs across the studied plant species (Table 1). It is noteworthy to mention that number of unique isomiR may be influenced by available sequencing data for the respective species. A total of 44,019 non-drift events were observed in non-templated isomiRs (Table 2). Among the drift events, 5A3D and 5D3A showed maximum abundance with 25,083 and 22,721, respectively (Table 2).

**Table 1:** Table showing the species enlisted in the Plant isomiR Atlas along with information on number of datasets, precursor, template, and non-templated isomiRs identified for each species and the most abundant isomiR in each species with the read count corresponding for that isomiR.

**Species**	**Dataset**	**Precursor**	**Templated**		**Non-templated**		**Unique**	**Most Abundant**	**Read Count**
			**Overlapped**	**Non-overlapped**	**Overlapped**	**Non-overlapped**	**Sequence**	
*Amborella trichopoda*	3	118	501	410	895	255	1,433	t-atr-isomiR-547	264,174
*Arabidopsis lyrata*	2	70	282	21	348	2	480	t-aly-isomiR-221	14,945
*Arabidopsis thaliana*	54	777	6,240	6,872	14,777	4,523	17,516	t-ath-isomiR-6620	17,495,442
*Brachypodium distachyon*	12	275	1,937	560	3,657	302	4,064	t-bdi-isomiR-1783	521,424
*Brassica rapa*	22	56	240	237	801	69	802	nt-bra-isomiR-882	403,865
*Carica papaya*	8	59	246	71	935	26	652	t-cpa-isomiR-138	386,874
*Citrus clementina*	5	5	10	16	25	10	44	nt-ccl-isomiR-39	25,425
*Citrus sinensis*	9	55	293	211	976	194	1,378	nt-csi-isomiR-2277	1,839,995
*Glycine max*	134	530	3,904	2,526	27,986	2,131	13,478	t-gma-isomiR-3578	29,661,609
*Gossypium raimondii*	4	123	143	220	545	510	1,037	nt-gra-isomiR-615	4,663,160
*Malus domestica*	14	158	697	385	3,024	159	1,384	t-mdm-isomiR-333	654,052
*Manihot esculenta*	6	138	630	348	2,633	113	1,451	t-mes-isomiR-389	1,204,480
*Medicago truncatula*	30	545	3,257	2,337	5,073	1,665	7,584	t-mtr-isomiR-2964	3,861,016
*Nelumbo nucifera*	11	97	487	249	1,097	241	1,133	nt-nnu-isomiR-1205	828,284
*Oryza sativa*	61	1,722	8,174	10,091	18,537	12,194	24,503	t-osa-isomiR-10418	4,331,151
*Populus trichocarpa*	4	344	992	410	2,458	279	1,017	t-ptc-isomiR-376	1,181,950
*Setaria italica*	6	133	776	1,007	1,018	270	1,725	t-sit-isomiR-974	734,334
*Solanum lycopersicum*	35	70	496	372	1,332	76	1,593	nt-sly-isomiR-1651	4,312,869
*Solanum tuberosum*	32	148	616	203	2,094	267	1,839	nt-stu-isomiR-3233	3,537,983
*Sorghum bicolor*	6	157	700	367	4,975	322	3,443	t-sbi-isomiR-639	2,613,920
*Triticum aestivum*	57	287	1,819	1,143	3,912	1,659	6,166	t-tae-isomiR-1932	10,779,136
*Vitis vinifera*	79	150	1,209	628	2,784	198	2,852	t-vvi-isomiR-1052	10,188,866
*Zea mays*	73	150	1,627	253	7,086	183	3,160	t-zma-isomiR-737	5,941,132

*The “overlapped” and “non-overlapped” indicated whether the isomiRs overlapped with canonical miRNAs in the same miRNA hairpin*.

**Table 2:** Table showing the location patterns of identified isomiRs.

**Species**	**Template**	**3A**	**3D**	**5A**	**5D**	**5A3A**	**5A3D**	**5D3A**	**5D3D**	**Non-drift**	**Non-overlapped**
*A. trichopoda*	Templated	39	81	22	52	7	163	119	18	–	410
	Non-templated	56	39	12	17	5	253	104	7	402	255
*A. lyrata*	Templated	23	69	17	41	2	76	49	5	–	21
	Non-templated	27	39	0	11	1	4	7	2	257	2
*A. thaliana*	Templated	441	394	351	234	274	2,047	2,418	81	–	6,872
	Non-templated	1,950	863	673	246	262	1,404	2,096	38	7,246	4,523
*B. distachyon*	Templated	130	261	83	187	37	509	702	28	–	560
	Non-templated	182	163	251	154	52	489	764	19	1,583	302
*B. rapa*	Templated	33	51	26	26	3	39	58	4	–	237
	Non-templated	84	33	58	16	11	23	62	3	511	69
*C. papaya*	Templated	21	60	20	63	0	46	23	13	–	71
	Non-templated	40	79	191	30	0	98	7	0	490	26
*C. clementina*	Templated	1	6	0	1	0	1	1	0	–	16
	Non-templated	0	6	0	0	0	0	1	0	18	10
*C. sinensis*	Templated	30	62	13	41	5	74	48	20	–	211
	Non-templated	227	169	180	30	12	65	30	8	255	194
*G. max*	Templated	354	467	305	243	149	1,119	1,156	111	–	2,526
	Non-templated	4,754	1,789	2,479	625	513	4,001	2,528	79	11,218	2,131
*G. aimondii*	Templated	8	12	5	13	2	26	77	0	–	220
	Non-templated	10	7	16	28	2	87	304	6	85	510
*M. domestica*	Templated	96	179	62	90	9	126	117	18	–	385
	Non-templated	302	183	132	85	9	25	39	1	2,248	159
*M. esculenta*	Templated	68	143	46	82	8	176	69	38	–	348
	Non-templated	240	411	47	93	1	210	27	7	1,597	113
*M. truncatula*	Templated	183	316	126	205	55	1,226	1,055	91	–	2,337
	Non-templated	429	294	163	59	77	1,474	950	5	1,622	1,665
*N. nucifera*	Templated	46	142	28	45	8	110	85	23	–	249
	Non-templated	90	149	50	31	7	80	52	6	632	241
*O. sativa*	Templated	380	696	271	536	165	2,879	2,865	382	–	10,091
	Non-templated	4,256	928	594	1,208	272	3,300	3,047	138	4,794	12,194
*P. trichocarpa*	Templated	109	157	61	105	10	248	271	31	–	410
	Non-templated	170	230	173	61	12	211	244	1	1,356	279
*S. italica*	Templated	65	112	35	62	10	172	309	11	–	1,007
	Non-templated	70	189	34	21	2	64	110	2	526	270
*S. lycopersicum*	Templated	61	113	33	77	2	89	100	21	–	372
	Non-templated	100	132	29	24	0	215	7	0	825	76
*S. tuberosum*	Templated	60	160	43	75	0	123	140	15	–	203
	Non-templated	94	164	50	37	0	244	178	3	1,324	267
*S. bicolor*	Templated	77	134	35	78	18	150	178	30	–	367
	Non-templated	1,389	415	168	567	27	677	137	21	1,574	322
*T. aestivum*	Templated	145	172	101	109	75	598	575	44	–	1,143
	Non-templated	295	231	156	67	148	961	798	15	1,241	1,659
*V. vinifera*	Templated	122	166	97	119	37	335	288	45	–	628
	Non-templated	436	324	186	62	48	194	62	10	1,462	198
*Z. mays*	Templated	186	273	149	190	79	363	311	76	–	253
	Non-templated	2,016	512	863	125	348	309	153	7	2,753	183
	Total	19,895	11,575	8,434	6,271	2,764	25,083	22,721	1,483	44,019

*3A, 3′ terminal addition; 3D, 3′ terminal deletion; 5A, 5′ terminal addition; 5D, 5′ terminal deletion; 5A3A, 5′ and 3′ terminal addition; 5A3D, 5′ terminal addition and 3′ terminal deletion; 5D3A, 5′ terminal deletion and 3′ terminal addition; 5D3D, 5′ and 3′ terminal deletion*.

Terminal modifications play an important role in defining the isomiRs and have been primarily linked with post-transcriptional uridylation events, cytidine additions or nucleotide addition that might regulate the 3′-5′ miRNA degradation as previously observed in *Larix leptolepis, A. thaliana* and other model plants (Zhang et al., 2013). Previously, template based terminal modification have been described as a measure of the base-line events that might indicate the isomiR biogenesis. We leveraged and focused on both the templated and non-templated isomiRs and interestingly observed that the non-templated isomiRs (Table S2) share the same pattern of terminal modification as templated isomiRs (Table S3). Table S3 details the observed terminal modification with detailed information on types of substitution such as internal single SNP are classified under SS, internal random SNPs are classified under MS, internal SNPs and terminal substitution are classified under CV, TS for having internal tandem SNPs, and 5 V/3 V for 5′- or 3′- end substitutions. Among the terminal modification cytidine addition was one the most dominant event in species such as *A. thaliana, B. distachyon, G. max, S. bicolor*, and *Z. mays* supporting the previous observations (Kulcheski et al., 2011; Zhang et al., 2013; Jacobs et al., 2016). Interestingly, we observed that the cytidine addition was also one of most prominent in the *A. trichopoda* suggesting that the cytidine addition for isomiR biogenesis might be an evolutionary conserved phenomenon (Table S3).

### Plant isomiR Atlas Visualization

**Figure 2:**
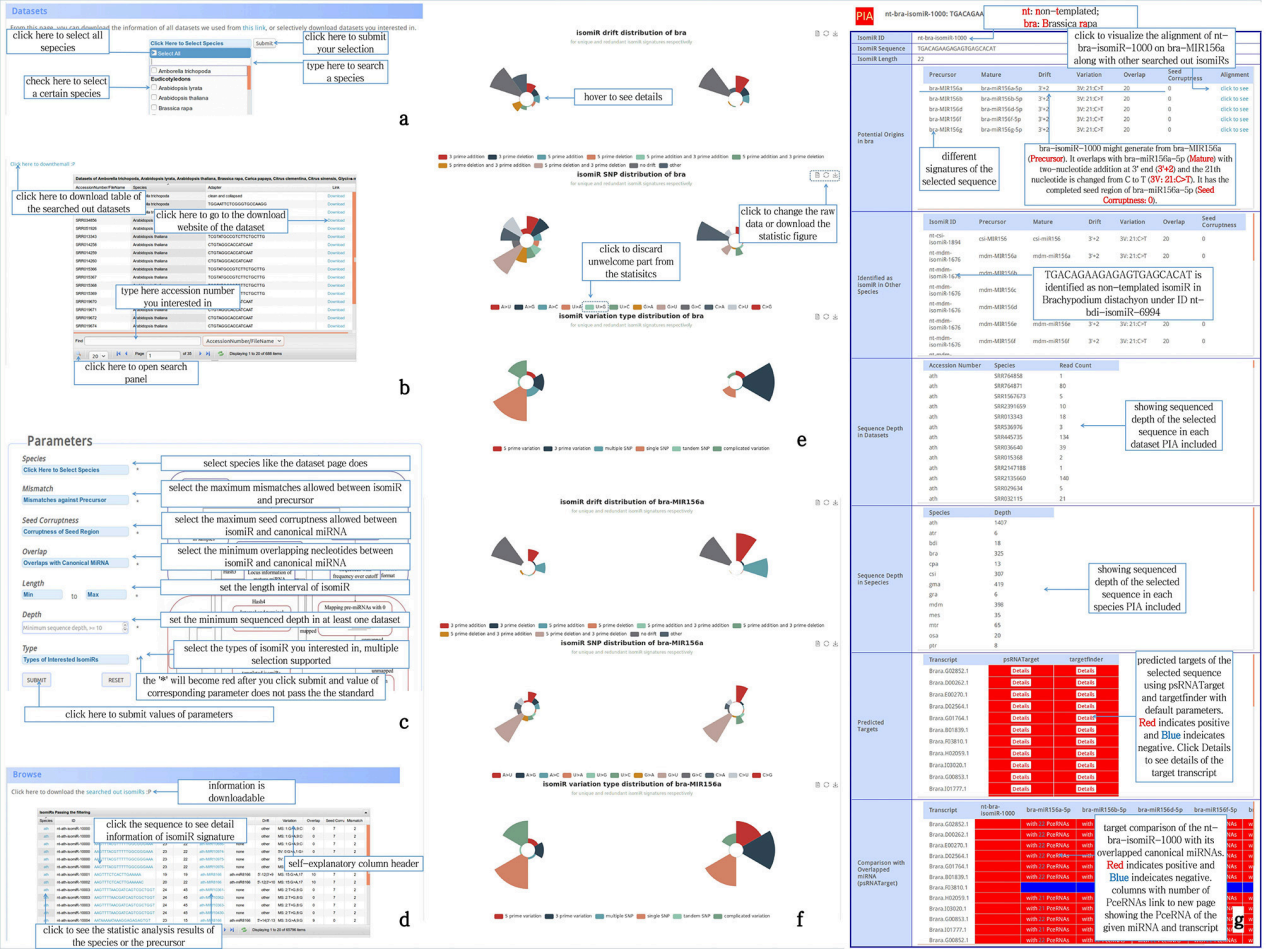
Schematic representation of browsing of Plant isomiR Atlas: **(a)** sub section shows the species selection according to species name or present an option to select all the species; **(b)** tabulated list of the datasets present for each species and also hyperlinked pages for the embedded datasets. Additionally, a final panel to allow user to search for particular accession; **(c)** the parameter page allows the user to define several parameters such as define species, mismatch, seed corruptness, overlap and minimum, and maximum length of isomiR with additional selection based on the depth of isomiR and the type of the isomiR; **(d)** Tabulated list of the isomiRs for the selected species with hyperlinks to see the isomiR associated information and does the isomiRs generated drift with the observed variation; **(e)** circular graphs showing the proportion of the variations observed in the species defined isomiRs; **(f)** circular graphs showing the proportion of the variations observed in the particular isomiR defined in the species; **(g)** isomiR classified page displaying multitude of information such as isomiR sequence, length, alignment of the isomiR with mature canonical miRNAs, potential origin of the isomiRs, isomiR overlap with other species, sequencing depth for the selected isomiR in the SRA datasets spanning for the selected species and across the other species in which the isomiRs shared the sequence conservation, target prediction for the selected isomiR using the psRNATarget and Targetfinder and a consensus overlap between the two target prediction algorithms.

Plant isomiR Atlas is an easy to browse web-repository of plant specific isomiRs and allows for the selection and visualization of profiled isomiRs along with the targets and canonical miRNAs information according to the species of interest. Among the features offered, users are allowed to browse the dataset information on the basis of species (Figure 2a). In the result page, tabulated dataset information, which is hyperlinked to the respective SRA accession used for the identification of isomiRs (Figure 2b) was shown. The parameter selection page offers several features such as overlap of the isomiR to the canonical miRNA, seed corruptness, mismatch type in case of non-templated isomiRs. Additionally, isomiRs can be further filtered based on the depth of the isomiRs and the types of the isomiRs classified on the basis of 5′- or 3′- end substitutions events (Figure 2c). Circular graphs and tabulated information for the drift events embedded within the Plant isomiR Atlas allows for the visualization of the associated drift events, nucleotide substitutions, and substitution patterns on the basis of species or on the basis of the selected miRNA precursors (Figures 2d–f).

Plant isomiR Atlas provides isomiR specific pages for each identified isomiRs (Figure 2g) with pre-defined features such as isomiR sequence, overlap with the canonical miRNAs, type of modifications and target predictions coming from the psRNATarget and TargetFinder. Diverse roles and functions of miRNAs have been well described previously and miRNAs interactions with the canonical target site in the mRNAs have been a subject of wide explorations. Recently, it has been shown that the miRNAs not only show lineage specific conserved functions but also diverge functions also, which increase the target repertoire of the plant miRNAs (Baldrich et al., 2018) It is noteworthy to highlight that these interactions have not only shown at the miRNAs level but also the level of siRNAs, which have alternative biogenesis pathway through the RdDM pathway. Irrespective of the biogenesis pathway, post-transcriptional regulatory role of miRNAs has been widely demonstrated ranging from the developmental to the functional landscape of plant abiotic and biotic stress. From the development point of view, regulatory role of the miR156 and their canonical post-transcriptional control of the SPL- family of genes have been widely illustrated. More details on capability of Plant isomiR Atlas can be found at the Help page of the database (http://www.mcr.org.in/isomir/help.php?pg=5).

In confluence with the recent reports, it is becoming increasingly evident that although the miRNA targets are evolutionary conserved for some families of miRNAs, they might however have additional lineage specific targets. From the recent miRNAs and siRNAs including the phasiRNAs studies, Ma et al. (2017) highlighted the functional divergence of the miR482/2118 family, which has in addition to the evolutionary conserved targets such as NB-LRR genes by showing additional functional targets such as protein kinases and zinc finger nucleases in *Litchi chinensis* Sonn. Taking into account these reports, it becomes imperative to explore the target site feasibility and also miRNA-target interactions. Ahmed et al. (2014) directed the conclusion that isomiRs coupled with canonical miRNAs can increase the functional target predictions. Taking this into account, we implement two target prediction algorithms in Plant isomiR Atlas *viz*. psRNATarget (Dai and Zhao, 2011) and TargetFinder (Fahlgren et al., 2007) independently and also consensus target predicted by both target prediction algorithms. Availability of these two target predictions allows the robust consensus selection of the predicted targets and allow for the identification of the functionally and evolutionarily conserved isomiRs with respect to the isomiR targets and will increase the target repertoire of the classified isomiRs for each species. For each isomiR, a cross-species conservation based on sequence conservation and also the concurrent read depth are provided. Recent studies have shown that genic miRNAs have different rates of evolutions such as the family targeting the chalcone 4 -O-glucosyltransferase (4 CGT) (Baldrich et al., 2018) and also the family specific miRNAs such as os-miR7695 in *O. sativa* (Baldrich et al., 2018) and miR-1510 in legumes (Baldrich et al., 2018). As the relationship between the canonical miRNAs and potential targets unravel with more diverse species, it is understandable that the corresponding isomiR diversity and their targets will eventually lead to better understanding of miRNAome. Taking this into account, Plant isomiR Atlas provides both the read based as well as the cross-species conservation of the identified targets for the isomiRs, which will accelerate the identification and development of the specific species isomiRs having shared or novel targets.

## Conclusion

To conclude, we have developed a large-scale plant specific isomiR repository, which features 23 plants species reresenting a total of 677 datasets and addresses both the templated and non-templated isomiRs profiling. Easy to browse plant isomiR atlas will accelerate the functional profiling of the templated and non-templated isomiRs and to understand the isomiR biogenesis and its conclusive regulatory roles. Understanding of the concurrent role of the isomiRs and the canonical miRNAs will contribute to increased understanding of non-coding miRNAome diversity and regulation genomics for strengthening the plant functional genomics.

## Author Contributions

GS conceived and designed the research. KY and GS analyzed the data. KY coded the Plant isomiR Atlas. SM hosted the Plant isomiR Atlas. XW took part in discussions. GS and KY wrote the manuscript.

### Conflict of Interest Statement

The authors declare that the research was conducted in the absence of any commercial or financial relationships that could be construed as a potential conflict of interest.
